# Effect of intraoperative blood transfusion on Treg and FOXP3 in patients with digestive tract malignancies and different ABO blood types

**DOI:** 10.1186/s12871-021-01330-9

**Published:** 2021-04-10

**Authors:** Yajun Liu, Junzhi Sun, Yun Xia, Michael R. Lyaker, Jianshe Yu

**Affiliations:** 1 Department of Anesthesiology, Affiliated Hospital of Inner Mongolia Medical University, Hohhot, Inner Mongolia China; 2 Department of Anesthesiology, The Ohio State University Wexner Medical Center, Columbus, OH 43210 USA

**Keywords:** Blood transfusion, ABO blood group, Treg, FOXP3, Immunity, Digestive tract, Malignant tumor

## Abstract

**Background:**

Blood transfusion can cause immunosuppression and lead to worse outcomes in patients with digestive tract malignancies; however, the specific mechanism behind this is not completely understood. One theory is that increased numbers of regulatory CD3^+^CD4^+^CD25^+^FOXP3^+^ T cells (Tregs) and forkhead box protein-3 mRNA (FOXP3) expression in the blood after transfusion contribute to these outcomes. The effect of blood transfusion on immune function in patients with different ABO blood types is variable. This study investigates the effect of intraoperative blood transfusion on the number of Tregs and the expression of FOXP3 in the blood of patients with different ABO blood types and digestive tract malignancies.

**Methods:**

Patients with digestive tract malignancies who underwent radical resection and received intraoperative blood transfusion were divided into four groups according to their blood types:blood group A, blood group B, blood group O and blood group AB (*n* = 20 for each group). Blood was collected from all patients before surgery, immediately after transfusion, 1 day after transfusion, and 5 days after transfusion. The number of Tregs was measured by flow cytometry. The expression of FOXP3 was detected by real time reverse transcription polymerase chain reaction (RT-PCR).

**Results:**

There was no significant difference in the number of Tregs or expression of FOXP3 mRNA among patients with different blood types before surgery. However, the number of Tregs and the expression of FOXP3 increased after blood transfusion in all blood type groups. This increase was especially evident and statistically significant on the first day after blood transfusion when compared with measures obtained before the surgery. Measures returned to the preoperative level five days after surgery. There were significant differences in the increase of Tregs and expression of FOXP3 among patients with different blood types. The greatest increase was seen in patients with blood group B and the least in blood group A.

**Conclusions:**

Intraoperative blood transfusion can lead to an increase in blood Tregs and FOXP3 expression in patients with digestive tract malignancies. Increases were greatest on the first day after surgery and differed among patients with different blood types. Increases were greatest in blood type B and least in blood type A.

## Background

Patients with malignant tumors of the digestive tract often have anemia because of tumor-associated bleeding, malnutrition, bone marrow suppression due to radiotherapy and chemotherapy, and intraoperative blood loss [1, 2]. Anemia not only affects the quality of life in these patients, but is also an independent risk factor for death [3]. Perioperative blood transfusion is commonly utilized to improve tissue oxygen delivery and perfusion, but transfusion also carries the risk of adverse effects [4]. Blood transfusion has been shown to induce immunosuppression in patients with cancer, expecially digestive tract cancer, then increase the risk of postoperative infections including lung and anastomotic infections, meanwhile increase early recurrence of malignancy and cancer-specific deaths, and reduce survival rates [5–8]. The complications can negatively affect patients’ prognosis. Previous studies have found that an increase in regulatory T cells (Tregs) and expression of their specific marker forkhead box protein-3 (FOXP3) after allogenic blood transfusion may be one of the mechanisms of immunosuppression [9]. However, the effect of blood transfusion on Tregs and FOXP3 in patients with digestive tract malignancies is unclear.

Treg generally refers to regulatory CD3^+^CD4^+^CD25^+^FOXP3^+^ T cells. These cells can have immunosuppressive effects through activation and expression of a variety of immune cells by cell-cell contact and cytokine-mediated mechanisms [10, 11]. FOXP3 is the specific marker of Tregs and is the key to their development, activation, and function [12, 13]. Deletion of FOXP3 can lead to the loss of Treg suppressive function on autoreactive T cells in scurfy (sf) mice. This has been shown to cause severe autoimmune reactions and death [14]. However, since it is an intracellular protein, the FOXP3/scurfin protein is not easy to detect. CD127 expression is inversely correlated with FOXP3 [15]; it is specifically expressed at a low level on the surface of Tregs and can be used as a biomarker for detecting them. Human ABO blood group substances are abundantly expressed on the surface of red blood cells and in various tissues, organs and body fluids [16]. ABO blood groups are associated with various diseases including tumors of the digestive system [17–21]. There are also differences in the levels of inflammatory factors and the incidence of transfusion reactions due to erythrocyte concentrates in patients with different ABO blood types [22]. The purpose of this study was to investigate the effect of blood transfusion on Tregs and FOXP3 expression in patients with malignant tumors of the digestive tract and different ABO blood types.

## Methods

This study was approved by the Ethical Committee of the Affiliated Hospital of Inner Mongolia Medical University, and written informed consent was obtained from all patients. The study selected patients at the Affiliated Hospital of Inner Mongolia Medical University from July 2018 to May 2019. Patients were divided into four groups according to their blood types, namely blood group A, blood group B, blood group O and blood group AB. The first 20 patients of each blood type who met selection criteria were assigned to each group. Peripheral venous blood was collected in EDTA anticoagulant tubes before surgery (T_0_), immediately after transfusion (T_1_), 1 day after transfusion (T_2_), and 5 days after transfusion (T_3_). The number of CD3^+^CD4^+^CD25^+^CD127^low^ Tregs and the expression of FOXP3 mRNA in the blood were detected.

### Inclusion and exclusion criteria

Inclusion criteria: (1) Patients diagnosed with a malignant tumor of the digestive tract by gastroscopy and needed radical resection (2) Preoperative hemoglobin < 100 g/L; (3) Aged 40–65 years old; (4) American Society of Anesthesiologists (ASA) physical status class II or III; (5) Body Mass Index (BMI) 18.5–24.9 kg/m^2^; (6) Rh blood group positive.

Exclusion criteria: (1) Severe lung or heart disease; (2) Presence of immune disease or recent use of immunosuppressive agents; (3) White blood cell count < 2 × 10 ^9^/L or platelet count < 80 × 10 ^9^/L; (4) Prothrombin time more than 3 s longer than normal control or activated partial thromboplastin time more than 10s longer than normal control; (5) Patients with a history of blood transfusion before surgery; no intraoperative blood transfusion was given; massive transfusion (transfusion of more than 4 units red blood cells within 1 h or 50% of total blood volume within 3 h) required during surgery or postoperative blood transfusion given. (6) patients with ABO non-identical transfusions in the perioperative period.

### Anesthetic technique

All patients received combined epidural and general anesthesia technique [23]. For the general anesthesia, anesthesia was induced by infusing sufentanil, etomidate and rocuronium and maintained by continuous infusing propofol, remifentanil and cisatracurium. For the epidural anesthesia, the patient was in the lateral decubitus position and the epidural intervertebral space was selected according to the surgical site. Once an epidural catheter was positioned, lidocaine 2% 5 ml was administered as a test dose and ropivacaine 0.25% 5 ml was supplemented every 40 min through the epidural catheter during the surgery. At the end of surgery, ramosetron 3 mg was intravenously administered. Postoperative analgesia was performed with patient-controlled epidural analgesia (PCEA).

### Blood transfusion method

All patients had a preoperative type and cross performed. According to the ASA “Practice Guidelines for Perioperative Blood Management” [24] and Chinese “Technical Specifications for Clinical Transfusion” [25], transfusion is not indicated when the hemoglobin concentration is above 10 g/dL, is indicated when the hemoglobin concentration is less than 7 g/dL, and should be guided by symptoms for patients with hemoglobin concentrations between 7 and 10 g/dL. The anesthesiologist determined the amount of blood transfused based on a comprehensive evaluation of the patient’s anemia, blood volume, blood loss, severity of shock, cardiopulmonary status and hemodynamic indicators.

### CD3^+^CD4^+^CD25^+^CD127^low^ Treg detection

Fifty μL of anticoagulated whole blood was thoroughly mixed in a test tube with 10 μL each of CD4-FITC/FL1-A (BD340133), CD25-APC/FL4-H (BD340938), CD127-PE/FL2-H (BD561028) monoclonal antibodies. The mixture was protected from light and maintained at room temperature for 15 min. Subsequently, 2 mL of a 1: 10 diluted FACS Lysing solution was added. The tube was incubated for 10 more minutes at room temperature while protected from light during lysis. The tube was centrifuged at 1500 rpm for 5 min and the supernatant removed. After adding 2 mL of phosphate buffer solution (PBS), the tube was again centrifuged at 1500 rpm for 5 min. The supernatant was removed and 500 μL PBS was added for detection. Detection was performed using a BD FACSCanto II flow cytometer, and analyzed by BD FACSDiva software to obtain the number of CD3^+^CD4^+^CD25^+^CD127^low^ Tregs.

### FOXP3mRNA detection

RNA was extracted using Trizol. The optical density (OD)260/OD280 ratios were measured with a micro-spectrophotometer to assess the RNA quality. A ratio between 1.8 and 2.0 met the experimental requirements. The total RNA concentration (μg/μL) = OD260 × 40 × 10^− 3^. First strand cDNA synthesis was performed using 10 μM oligo (dT)18 primer and Hiscript reverse transcriptase. Reaction conditions were 5 min at 25 °C, 15 min at 50 °C, 5 min at 85 °C, and 10 min at 4 °C. The resulting cDNA was diluted twice before proceeding with the PCR reaction. The relative DNA amount of FOXP3 mRNA was determined using SYBR Green Master Mix, and the internal reference gene GAPDH was measured in each sample. The PCR reaction conditions were 40 cycles at 50 °C for 2 min, 95 °C for 10 min, 95 °C for 30 s, and 60 °C for 30 s. The primer sequences are as follows: FOXP3 upstream primer 5′-CATTCCCAGAGTTCCTCCACA-3′, downstream primer 5′-CATTGAGTGTCCGCTGCTTC-3′; the internal reference gene GAPDH upstream primer 5′-TCAAGAAGGTGGTGAGACAGG-3′, and downstream primer 5′- TCAAAGGTGGAGGAGTGGGT-3′. Triplicate wells were conducted for each specimen, and the final data was analyzed for changes in the relative expression level of FOXP3 mRNA using the 2^-△△Ct^ method [26, 27].

### Statistical analysis

The data were analyzed by using SPSS18.0. Quantitative data were expressed as mean ± standard deviation ($\overline{x}\pm s$), and discrete data were expressed as numbers. Comparison within groups at different time points was performed by repeated measures Analysis of Variance (ANOVA), and comparison between different groups at the same time points was performed using the Bonferroni test. Discrete data were compared using chi-square test or Fisher’s exact test. *P* < 0.05 was considered as statistically significant.

### Sample size

The formula $\mathrm{n}=4\;{\left[\frac{Z_{a/2}+{Z}_{\beta }}{1\mathrm{n}\;\frac{\left(1+p\right)}{1-p}}\right]}^2+3$ was used to estimate sample size based on the correlation coefficient test. The correlation coefficient of 0.7 was substituted into the formula; assuming two-sided α = 0.05, β = 0.10, then the sample size would be *n* ≈ 17. Therefore at least 17 cases were required for each group.

## Results

### Patient demographics

There were no significant differences among different blood groups with regard to age, sex, BMI, tumor type, treatment history, preoperative hemoglobin, operative time, operative technique or volume of intraoperative blood transfusion (*P* > 0.05). (Table 1).

**Table 1:** Patient demographics

	A	B	O	AB	*F/****χ***^**2**^	*P*
Number of patients	20	20	20	20		
Age (yr)	53.45 ± 5.29	53.55 ± 5.9	54 ± 5.52	54.95 ± 6.68	0.272	0.845
Sex (*n*)					0.158	0.984
Male	8	8	7	8		
Female	12	12	13	12		
BMI (kg/m2)	21.48 ± 1.98	22.47 ± 1.38	21.79 ± 1.78	21.8 ± 1.7	1.178	0.324
Tumor type (*n*)					5.348	0.803
Rectal cancer	3	2	3	3		
Colon cancer	8	6	9	11		
Gastric cancer	8	11	8	6		
Esophageal cancer	1	1	0	0		
Treatment history (*n*)					0.213	0.975
No	8	7	7	8		
Chemoradiation	12	13	13	12		
Preoperative hemoglobin (g/L)	83.95 ± 8.42	84.45 ± 10.2	83.25 ± 8.74	83.55 ± 7.88	0.690	0.976
Operative time (min)	200 ± 30.96	213.7 ± 40.98	218.95 ± 25.9	226.55 ± 29.57	2.394	0.075
Operative technique (*n*)					0.595	0.898
Laparotomy	8	6	8	7		
Peritoneoscopy	12	14	12	13		
Blood transfusion volume (ml)						
RBC	425.0 ± 99.9	456.0 ± 113.1	440.0 ± 96.9	450.5 ± 113.3	0.331	0.803
Plasma	474.5 ± 154.5	442.0 ± 140.1	506.0 ± 167.4	514.0 ± 165.9	0.872	0.460

Data are expressed as number or mean ± standard deviation. *A* blood group A, *B* blood group B, *AB* blood group AB, *O* blood group O, *BMI* Body–mass index, *RBC* red blood cells, *T*_*0*_ before surgery, *T*_*1*_ immediately after transfusion, *T*_*2*_ 1 day after transfusion, *T*_*3*_ 5 days after transfusion

### Flow cytometry assay comparison of CD3^+^CD4^+^CD25^+^CD127^low^Tregs at different time points for different blood types

**Fig. 1:**
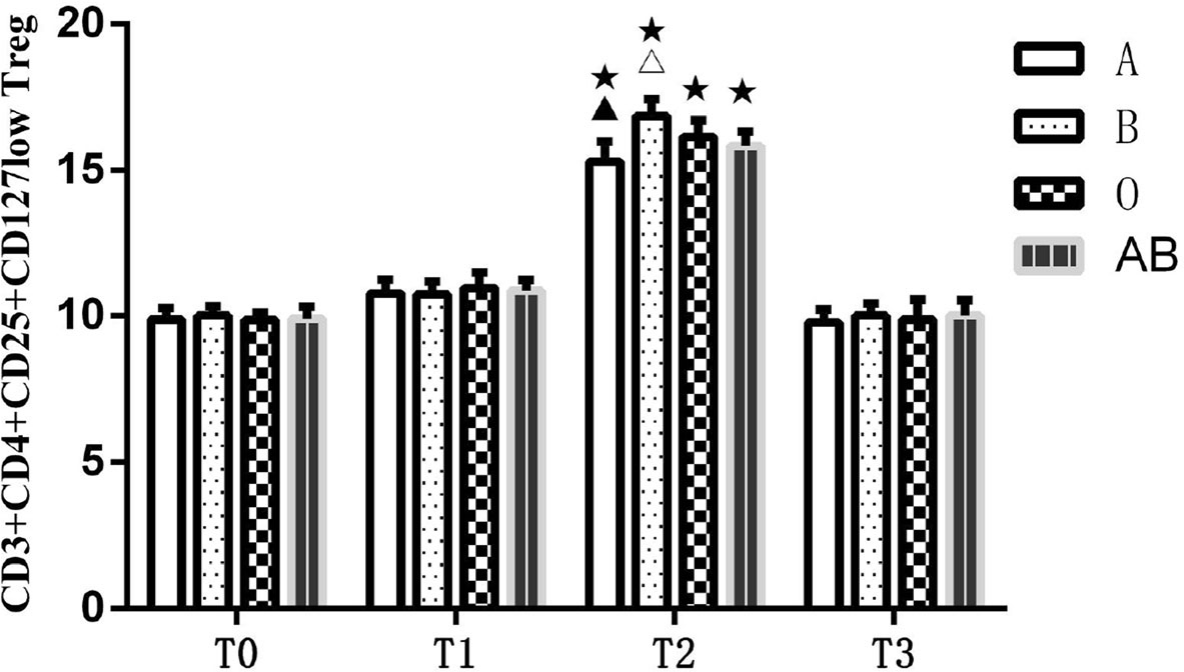
Comparison of CD3^+^CD4^+^CD25^+^CD127^low^ Tregs at different time points in different blood types. A, blood group A; B, blood group B; O, blood group O; AB, blood group AB. T_0_, before surgery; T_1_, immediately after transfusion; T_2_, 1 day after transfusion; T_3_, 5 days after transfusion. Compared with T_0_, ★*P* < 0.05; compared with blood group B, O, AB, ▲*P* < 0.05; compared with blood group A, O, AB, △*P* < 0.05

**Fig. 2:**
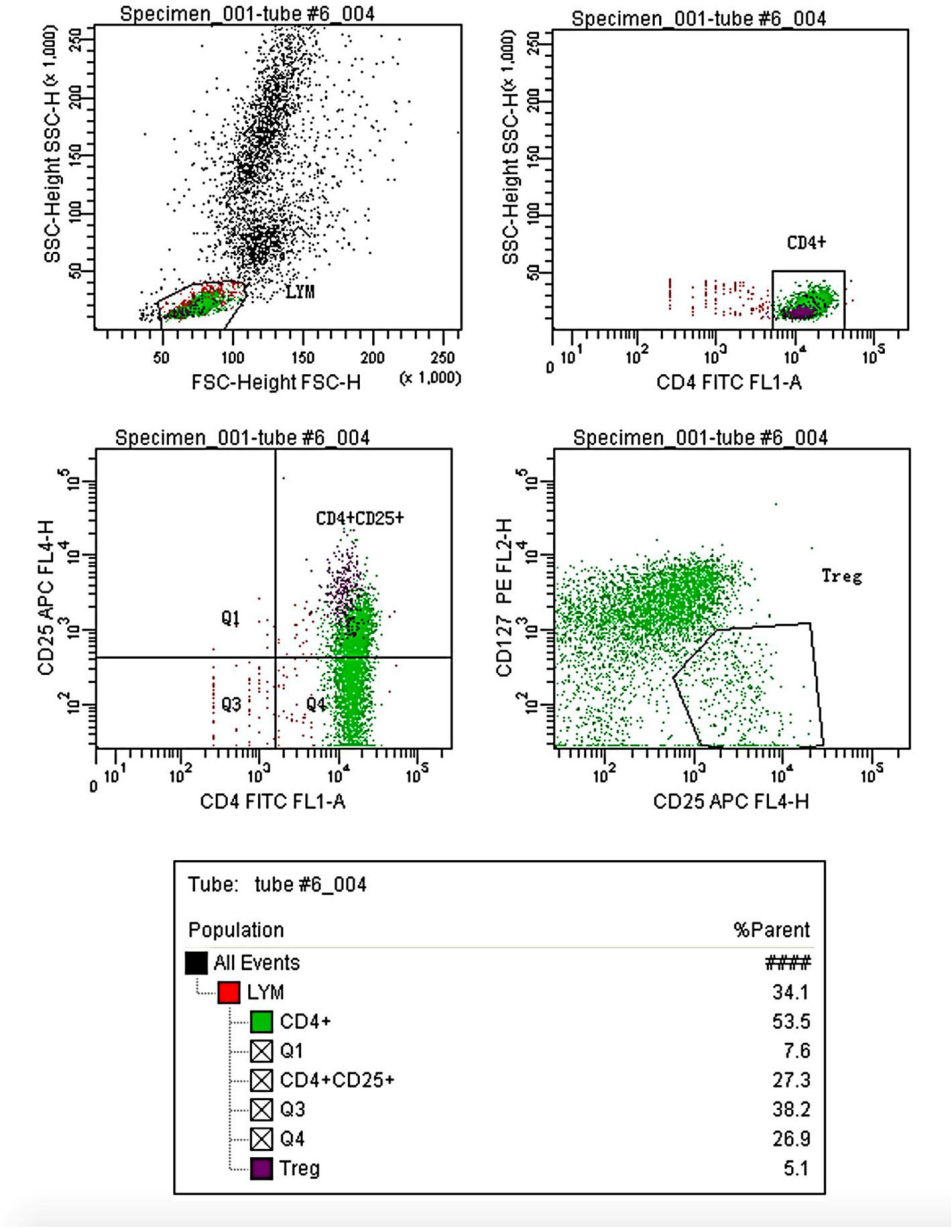
Flow cytometry image. Flow cytometric gating scheme for Treg: Starting from lymphocytes by FSC and SSC gating, cells were further gated on CD4^+^ and CD25^+^, then CD127^low^ was used to define Treg population as CD4^+^CD25^+^CD127^low^ T lymphocytes, gating of this marker was based on CD4^+^ T lymphocytes

Between-group and within-group ANOVA was performed on CD3^+^CD4^+^CD25^+^CD127^low^ Tregs in the four groups of patients with different blood types (Table 2) (Fig. 1). The results showed that there was no significant difference in peripheral blood CD3^+^CD4^+^CD25^+^CD127^low^ Treg numbers among patients with different blood types at T_0_ (*P* > 0.05). The number of Tregs increased at T_1_, but there was no statistically significant difference from T_0_ (*P* > 0.05). At T_2_ Treg numbers were significantly increased compared with T_0_ (*P* < 0.05). At T_3_ Treg numbers returned to preoperative levels, and there was no significant difference compared with T_0_ (*P* > 0.05). The degree of increase in CD3^+^CD4^+^CD25^+^CD127^low^ Treg numbers after blood transfusion was different among patients with the four different blood types. The highest increase was seen in blood group B and the least in blood group A; This difference was statistically significant (*P* < 0.05) at T_2_. The results of CD3^+^CD4^+^CD25^+^CD127^low^ Treg detection by flow cytometry are shown in Fig. 2.

**Table 2:** Comparison of CD3^+^CD4^+^CD25^+^CD127^low^ Tregs at different time points in different blood types

Group	*N*	T0	T1	T2	T3
A	20	9.88 ± 0.39	10.77 ± 0.49	15.29 ± 0.68^★▲^	9.77 ± 0.45
B	20	10 ± 0.32	10.74 ± 0.43	16.84 ± 0.58^★△^	10.01 ± 0.41
O	20	9.85 ± 0.27	10.96 ± 0.53	16.14 ± 0.55^★^	9.88 ± 0.68
AB	20	9.91 ± 0.4	10.86 ± 0.38	15.83 ± 0.50^★^	10.00 ± 0.54
F		0.711	0.912	25.053	0.910
*P*		0.548	0.439	0.000	0.440

Data are expressed as mean ± standard deviation. *A* blood group A, *B* blood group B, *AB* blood group AB, *O* blood group O, *T*_*0*_ before surgery, *T*_*1*_ immediately after transfusion, *T*_*2*_ 1 day after transfusion, *T*_*3*_ 5 days after transfusion. Compared with T_0_, ★*P* < 0.05; compared with blood group B, O, AB, ▲*P* < 0.05; compared with blood group A, O, AB, △*P* < 0.05

### Comparison of FOXP3mRNA at different time points in different blood types

**Fig. 3:**
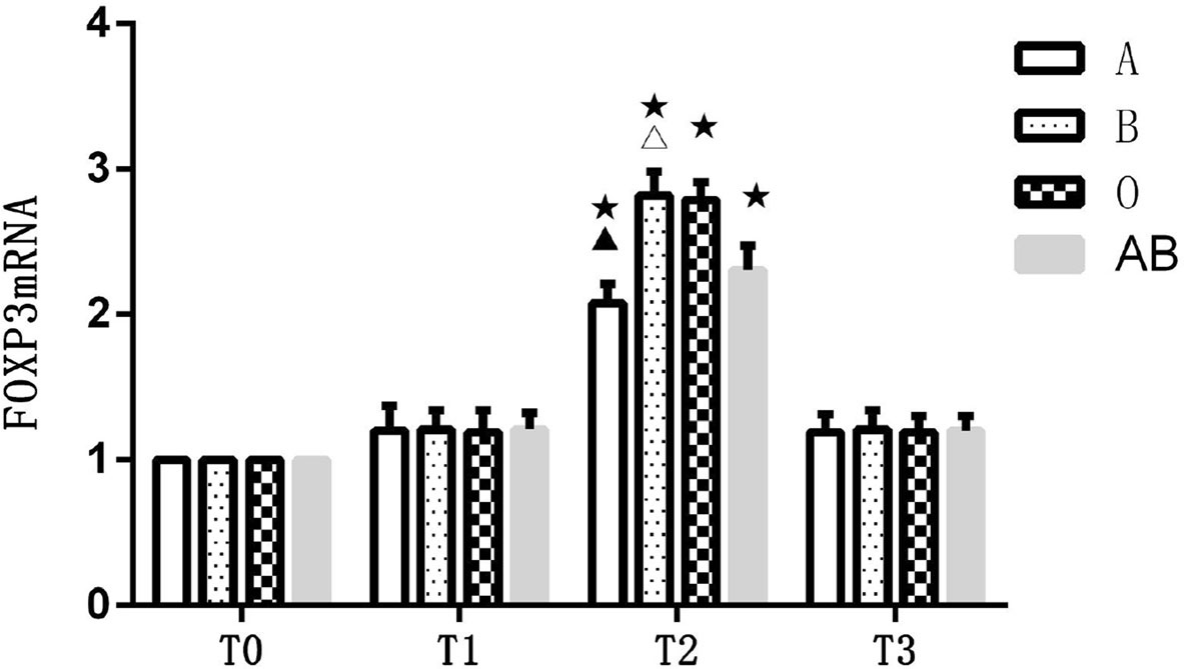
Comparison of FOXP3mRNA at different time points in different blood types. A, blood group A; B, blood group B; O, blood group O; AB, blood group AB. T_0_, before surgery; T_1_, immediately after transfusion; T_2_, 1 day after transfusion; T_3_, 5 days after transfusion. Compared with T_0_, ★fold-change > 2; compared with blood group B, O, AB, ▲*P* < 0.05; compared with blood group A, O, AB, △*P* < 0.05

The FOXP3mRNA expression at T_1_, T_2_ and T_3_ relative to T_0_ in all patients was calculated by relative quantitative calculation using 2^-△△Ct^ formula; the fold-change of absolute expression > 2 was considered to be statistically significant. The FOXP3mRNA expression at T_0_ among all patients with different blood types was 1_._ Between-group and within-group ANOVA was performed on FOXP3mRNA in the four groups of patients with different blood types (Table 3) (Fig. 3). Results showed that compared with T_0_, expression of FOXP3mRNA in the peripheral blood of patients with all four blood types began to increase at T_1_ (fold-change < 2), peaked at T_2_ (fold-change > 2) and then decreased to preoperative levels at T_3_ (fold-change < 2). The degree of increase in FOXP3mRNA expression was different after blood transfusion among patients within the four blood types. The highest increase was seen in blood group B and the lowest was seen in blood group A. This difference was statistically significant (*P* < 0.05).

**Table 3:** Comparison of FOXP3mRNA at different time points in different blood types

Group	*N*	T0	T1	T2	T3
A	20	1.00	1.2 ± 0.17	2.08 ± 0.13^★▲^	1.19 ± 0.12
B	20	1.00	1.21 ± 0.13	2.82 ± 0.16^★△^	1.21 ± 0.13
O	20	1.00	1.19 ± 0.15	2.79 ± 0.12^★^	1.19 ± 0.11
AB	20	1.00	1.21 ± 0.11	2.31 ± 0.16^★^	1.2 ± 0.1
F			0.095	125.993	0.148
*P*			0.963	0.000	0.931

Data are expressed as mean ± standard deviation. *A* blood group A, *B* blood group B, *AB* blood group AB, *O* blood group O, *T*_*0*_ before surgery, *T*_*1*_ immediately after transfusion, *T*_*2*_ 1 day after transfusion, *T*_*3*_ 5 days after transfusion. Compared with T_0_, ★fold-change > 2; compared with blood group B, O, AB, ▲*P* < 0.05; compared with blood group A, O, AB, △*P* < 0.05

## Discussion

Our results found that intraoperative blood transfusion may lead to an increase in Treg and FOXP3 in the blood of patients with digestive tract malignancies. For the patients with different ABO blood types, there was increase in Treg and FOXP3, but differed in degree. The patients with blood type B had the highest increase and those with blood type A showed the lowest increase.

The immune system plays a key role in the body’s defense against malignancy. While immune function in patients with malignant tumors is often impaired, the specific mechanism is not clear. Numerous studies have demonstrated that Tregs and FOXP3 are increased in the peripheral blood and tumors of patients with digestive tract malignancies and are positively correlated with tumor stage [28, 29]. In addition, increases of Tregs and FOXP3 in patients with digestive tract malignancies are associated with a poor prognosis because of an association with tumor immune escape [30]. Tregs are mainly produced by the thymus (tTregs), but can also be produced in the periphery (pTregs) or induced in cell culture (iTregs) [31]. Tregs can directly or indirectly inhibit the activation of natural killer (NK) cells, cytotoxic T lymphocytes, monocytes, B cells, inhibit the expression of macrophages, and regulate the expression of T helper type 1 (Th1) and type 2 (Th2) cells. This occurs through direct cell-to-cell contact [10], secretion of multiple inhibitory cytokines such as transforming growth factor-β (TGF-β), interleukin (IL)-10, and through inhibiting the production of interferon-γ (IFN-γ) and tumor necrosis factor-α (TNF-α) [15]. These actions cumulatively exert an immunosuppressive effect on the body’s defenses [11]. FOXP3 is a member of the forkhead/winged-helix transcription factor family. It is specifically expressed on Treg without being affected by activation status and is distinct from other molecular markers of Tregs such as CD25, CD45RB, cytotoxic T lymphocyte-associated antigen 4 (CTLA-4), and glucocorticoid-induced tumor necrosis factor receptor (GITR). FOXP3 is the key to the development, activation and function of Tregs [12, 13]. FOXP3 may also participate in the regulation of Tregs through the dual roles of transcription repressor and activator [10]. Only changes in FOXP3 on thymus Tregs can affect Treg numbers in peripheral blood [32].

Patients with digestive tract malignancies often have pre-existing anemia and require intraoperative blood transfusion [2]. Blood transfusion can increase the risk of metastasis and recurrence, as well as the incidence of complications such as local infection, pneumonia, and sepsis [5, 6]. Factors such as autologous and allogeneic blood transfusion [33, 34], use of different blood products and blood storage times [35], have been commonly studied with regard to immunosuppression. However, the mechanism of immunosuppression from blood transfusion remains uncertain [9, 36]. Proposed mechanisms include: enhanced secretion of cytokines such as prostaglandins, thromboxane, growth factors, nonpolar lipids and pro-inflammatory lysophosphatidylcholines [37–39]; down-regulated expression of Th1 type cytokines (IL-2, IL-12, IFN-γ and TNF-γ); up-regulated expression of Th2 type cytokines (IL-4, IL-5, IL-6 and IL-10) [38]; increases in the population of Tregs [39] and formation of microparticles [40] among others. At present, few studies have explored the mechanism of blood transfusion induced immunosuppression in patients with digestive tract malignancies. Transfusion-induced up-regulation of Tregs and FOXP3 may be an important mechanisms of immunosuppression after blood transfusion in patients with digestive tract malignancies [9]. The present study shows that intraoperative blood transfusion may temporarily increase the number of Tregs and the expression of FOXP3 in this patient population. This was especially evident on postoperative day 1 and resolved by postoperative day 5. Given the known role of Tregs in modulating immune function, our finding supports that increased blood levels of Treg and FOXP3 may play an important role in the immunosuppression seen with blood transfusion. Since, the observation time of this study was short and the sample size was relatively small, these results still need further investigation to confirm.

The ABO blood group antigen system was discovered a century ago. Human ABO blood group system consists of three alleles (A, B, O) and four phenotypes. Studies have shown that ABO blood groups are associated with various diseases such as cardiovascular disease [20] diabetes [21], and malignant tumors including those of the digestive tract [17–19]. The incidence of transfusion reactions and levels of inflammatory cytokines also differs among patients with different ABO blood groups [22]. However, the biological and functional role of the ABO blood group antigen system remains a mystery [41]. This study showed that the degree of increase in Tregs and FOXP3 after blood transfusion was different in patients with different ABO blood types. This increase was highest among patients with blood type B and least in patients with blood type A. This finding suggests that the degree of immunosuppression in patients with digestive tract malignancies and different ABO blood types may also vary. Further studies with larger numbers of patients are still needed to confirm this finding. We speculate that the increase in the number of Tregs and FOXP3 expression in these patients with blood type B may be related to the lack of the A antigen on erythrocytes and the increase of anti A antibodies in the plasma, but this study has not found the same trend in patients with blood type O, so the mechanisms should be further studied in future. The correlation between different ABO blood types and immunosuppression after blood transfusion might has significant implications in clinical blood use. For example, patients with blood type B and a high risk of requiring perioperative transfusion may benefit from a comprehensive, individualized blood management protocol to reduce the need for blood transfusion as much as possible.

### Limitations

Our study documented an excessive of plasma, which was different from many transfusion practice. First, the usage of colloids was strictly limited by our hospital. Second, the patients in our study received epidural anesthesia combined with general anesthesia, this anesthesia method always lead to hypotension during the intraoperative period. Therefore, fresh frozen plasma was used as a common therapy for these patients to maintain intraoperative hemodynamic stability in our center. Furthermore, we just tested the biomarkers and found that there were significant differences between the ABO blood groups. In future, we should follow up the patients and observe whether the incidence of complications and reoccurrence rate are different between the ABO blood groups.

In conclusion, these findings suggest that the degree of immunosuppression after blood transfusion in patients with digestive tract malignancies and different ABO blood types might be different as well. While these findings still need to be confirmed by larger trials, such investigations may lead to individualized blood management for patients with different ABO blood types. Our findings suggest that the degree of immunosuppression after blood transfusion in patients with digestive tract malignancies and different ABO blood types might be different as well. While these findings still need to be confirmed by larger trials, such investigations may lead to individualized blood management for patients with different ABO blood types.

## Conclusions

Intraoperative blood transfusion can lead to an increase in blood Tregs and FOXP3 expression in patients with digestive tract malignancies. Increases were greatest on the first day after surgery and differed among patients with different blood types. Increases were greatest in blood type B and least in blood type A.

## Data Availability

The datasets used and analyzed during the current study are available from the corresponding author on reasonable request.

## Abbreviations

Tregs: Regulatory CD3 + CD4 + CD25 + FOXP3+ T cells
FOXP3: Forkhead box protein-3
RT-PCR: Real time reverse transcription polymerase chain reaction
ASA: American Society of Anesthesiologists
BMI: Body Mass Index
GA: General anesthesia
P_ET_CO_2_: End-tidal carbon dioxide tension
PCEA: Patient-controlled epidural analgesia
PBS: Phosphate buffer solution
OD: Optical density
ANOVA: Analysis of Variance
NK cells: natural killer cells
Th1: T helper type 1
Th2: T helper type 2
TGF-β: Transforming growth factor-β
IL: Interleukin
IFN-γ: Interferon-γ
TNF-α: Tumor necrosis factor-α
CTLA-4: Cytotoxic T lymphocyte-associated antigen 4
GITR: Glucocorticoid-induced tumor necrosis factor receptor
